# Urinary Tract Infections in the Kingdom of Saudi Arabia, a Review

**DOI:** 10.3390/microorganisms11040952

**Published:** 2023-04-06

**Authors:** Idris Sula, Mateq Ali Alreshidi, Najah Alnasr, Ahmad M. Hassaneen, Nazmus Saquib

**Affiliations:** 1 College of Applied Sciences, Sulaiman Al Rajhi University, P.O. Box 777, Al Bukayriah 51941, Saudi Arabia; idris.sula111@gmail.com (I.S.); a.hassaneen@sr.edu.sa (A.M.H.); 2 Department of Medical Laboratory Sciences, College of Applied Sciences, Sulaiman Al Rajhi University, P.O. Box 777, Al Bukayriah 51941, Saudi Arabia; ma.alreshidi@sr.edu.sa (M.A.A.); najahalnasr@gmail.com (N.A.); 3 Clinical Pathology Department, Faculty of Medicine, Zagazig University, Zagazig 44519, Egypt; 4 College of Medicine, Sulaiman Al Rajhi University, P.O. Box 777, Al Bukayriah 51941, Saudi Arabia

**Keywords:** UTI, urinary tract infections, uropathogens, Saudi Arabia, KSA

## Abstract

Urinary tract infections (UTIs) are among the most common infections and are associated with an increased rate of antimicrobial resistance in Saudi Arabia. Better knowledge of the most common pathogens and their antimicrobial resistance patterns will be useful for creating new treatment guidelines. PubMed, Web of Science, Scopus, and Google Scholar were searched using suitable keywords to identify UTI publications until November 2022. Eligible studies were selected and analyzed. A total of 110 records were found, but only 58 articles were analyzed. Most studies were retrospective, and just a few were cross-sectional or prospective. The majority of the studies were conducted in the central region followed by the Eastern region. *Escherichia coli* and *Klebsiella* spp. were the most common pathogens. There was a significant resistance rate against co-trimoxazole and ciprofloxacin. On the other hand, amikacin was one of the most effective antibiotics. Overall, only a few studies have been published on UTIs in Saudi Arabia. Moreover, not all regions have been represented, so the full scope of the issue is unknown. UTIs are still a major problem, and resistance has developed against commonly used antibiotics. Thus, large epidemiological studies are needed to battle the rapid emergence of antimicrobial resistance.

## 1. Introduction

Urinary tract infections (UTIs) are one of the most common types of infections [1]. They tend to be much more common in women; around 60% of women can expect to experience at least one UTI in their lifetime [2,3]. UTIs remain a burden for the healthcare system in the Kingdom of Saudi Arabia (KSA); they account for 10% of all infections in the country, and they stand as the second most common reason for emergency department admissions [2,3]. Around 4% of UTI patients are admitted to the hospital for further treatment. Another issue is readmission; around 10% are readmitted within one week of their discharge, and one of the main reasons is ineffective treatment [3]. Similar problems are faced around the world, as has been reported by several studies [4,5].

UTIs have usually been treated with broad-spectrum antibiotics. However, the treatment is often started without taking into consideration the bacterial culture or antimicrobial sensitivity patterns, which has led to the development of antimicrobial resistance worldwide. Nowadays, there is an alarming rate of antimicrobial resistance, leading to multidrug-resistant (MDR) bacteria [6]. Antimicrobial susceptibility patterns of the same bacteria can vary according to geographic location [5]. The Infectious Diseases Society of America recommends that regional surveillance should be conducted to monitor changes in the susceptibility of uropathogens in specific regions [7].

According to the European Union, the rate of human deaths related to antibiotic-resistant bacterial infections is approximately 25,000 per year, and two-thirds of these infections are due to Gram-negative bacteria [6,8]. One of the key factors contributing to the increased rate of antimicrobial resistance is overdiagnosis, which results in the overuse of antibiotics that might be unnecessary [9,10]. Hence, the diagnosis by urine culture is a necessity, especially for complicated UTIs, as it will confirm the infection and provide the physician with an antimicrobial pattern for that particular pathogen [4].

The main challenge in prescribing the treatment after confirming an infection by culture is time. According to the Saudi National Antimicrobial Therapy Guidelines, asymptomatic bacteriuria can be treated with empirical therapy without confirmation from a positive culture. Antimicrobials suich as nitrofurantoin, co-trimoxazole, ertapenem, and imipenem are among the first line of defense [10,11]. However, symptomatic bacteriuria should be treated after a confirmed microbiological culture, and it takes 2 to 3 days to receive a culture report in the microbiology lab [9,10]. International guidelines from the World Health Organization (WHO) suggest using nitrofurantoin and co-trimoxazole as the first line of defense. These treatments are also highly emphasized in the local guidelines [2,3]. Estimating the prevalence of the most commonly isolated pathogens and their antimicrobial resistance patterns is crucial for every hospital to avoid the emergence of new antibiotic resistance. Knowledge of the local antimicrobial sensitivity pattern is periodically required to plan an updated treatment regimen [12].

UTIs are very common, and bacterial antimicrobial resistance is emerging around the world, particularly in KSA. Most studies have been retrospective and descriptive in nature [13,14,15,16,17,18,19,20,21,22,23,24,25,26,27,28,29,30,31,32,33,34,35,36,37,38,39,40,41]. Most studies have tried to raise awareness of the emergence of antimicrobial resistance, but many of the data available are outdated [16,22,23,25,26,31,39,40,41,42,43,44]. Hence, periodic updates are needed to track this emergence. Moreover, no review papers have been published on this matter in KSA. This review is important as it will increase awareness of UTIs and antimicrobial resistance patterns in KSA [40,42,43,44]

As antimicrobial susceptibility patterns change rapidly, new resistant strains are emerging. Our review focused on the most common pathogens and their antibiotic susceptibility patterns. We hope this review can raise awareness among local healthcare professionals about emerging resistant strains, help them in their decisions to prescribe antibiotics, and be a tool to prevent and control the spread of resistant strains in the future.

## 2. Materials and Methods

### 2.1. Literature Review and Data Sources

The articles were found by searching PubMed, Web of Science, Scopus, and Google Scholar. All the studies published through November 2022 were included. Search keywords included urinary tract infection, uropathogen, UTI, Gram-negative bacteria, Gram-positive bacteria, antibiotics, antibiotic resistance, KSA, and Saudi Arabia. Additionally, national journals (*Saudi Medical Journal* and *Annals of Saudi Medicine*) and the reference lists of eligible articles were searched to identify additional published studies.

### 2.2. Eligibility Criteria

All published studies that (1) examined urinary tract infections and (2) were conducted in the Kingdom of Saudi Arabia were eligible for review (i.e., inclusion criteria). In addition, eligible studies had to include isolation and identification of UTI-causing uropathogens based on standard bacteriological methods following the Clinical Standards Laboratory Institute (CSLI) guidelines and using the approved automated systems or manual methods. Publications about UTIs that did not stem from primary research (e.g., opinion and letters to the editor), or were from conference proceedings or abstracts, were not included (i.e., exclusion criteria). All the studies were full-text and published in English.

### 2.3. Search Outcomes

**Figure 1:**
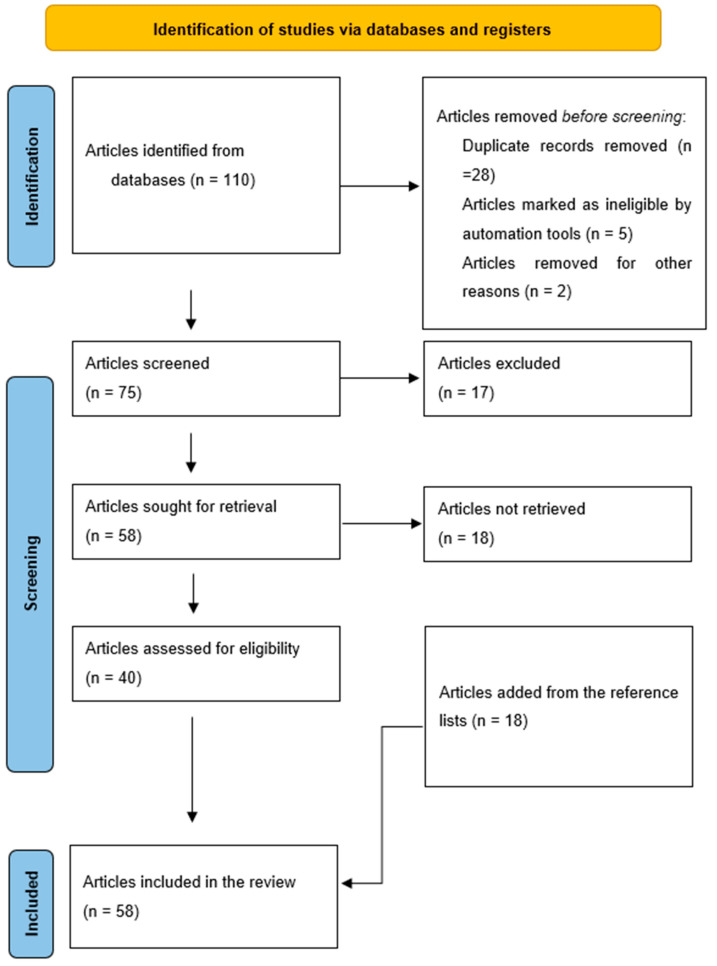
Flowchart of eligible articles.

A total of 110 records were found. These produced 95 unique records after 28 duplicate articles were removed through a careful read of the study titles. Manuscripts were screened carefully, and only 40 met the inclusion criteria. An additional 18 articles were found while scanning eligible articles and their references, bringing the total to 58 articles for analysis (Figure 1).

### 2.4. Data Extraction Process and Analysis

From each included study, the following data were abstracted: (1) authors’ names, (2) publication year, (3) study design, (4) sample age and size, (5) study population, (6) location, and (7) main findings. Initially, the data of the included studies were charted by one co-author and then reviewed independently by the lead and senior authors. The main findings of the eligible articles were categorized under broad themes.

## 3. Results

### 3.1. General Description of the Studies 

The majority of the studies were retrospective studies, and just a few were cross-sectional or prospective. The first study was published in 1988, and the following decades (i.e., 1991–2000 and 2001–2010) saw an approximately equal proportion of publications, whereas the last decade (i.e., 2011–2021) saw the most publications. Most studies were conducted in the central region, which includes Riyadh and other nearby cities, followed by the eastern region, Jeddah, and the Al Qassim region. Other regions had one or two publications per region. All age groups were included, from neonates to older adults (aged from 0 to +90 years). The sample sizes ranged from 82 [22] to 49,779 [27] participants (Table 1).

### 3.2. UTI in Diabetic Patients

Different comorbidities such as diabetes and pregnancy are associated with complications such as urinary tract infections [69]. Five studies specifically mentioned the UTI prevalence in diabetic patients [13,14,25,46,65]. A study conducted in Riyadh found that diabetes was one of the main causes of complicated UTIs, which extends beyond the bladder to the upper urinary system [13]. In another study, it was reported that 25.3% of the general diabetic population experienced UTIs [13]. Female patients were at much higher risk compared to men, even among patients who did not have diabetes or other chronic conditions [13,25]. There were other risk factors that were associated with an increased risk for developing UTI among diabetic patients, such as hypertension (*p* = 0.006), microalbuminuria (*p* = 0.031), insulin therapy (*p* < 0.001), and a body mass index (BMI) greater than 30 kg/m^2^ (*p* < 0.001), all of which are complications of diabetes [14,46]. Other factors such as age, diabetes type, duration of diabetes, and HbA1C levels did not significantly increase the risk of developing a UTI [46]. Diabetic patients are at high risk of asymptomatic pyuria, bacteriuria, and upper urinary tract infections. One of the main factors is glucosuria, as glucose is used as a source of energy for bacteria, thus creating a suitable environment for bacteria to replicate at a high rate; it is also related to neutrophil dysfunction [13,25]. 

The most common isolated pathogens were *Escherichia coli*, *Pseudomonas*, and *Staphylococcus hominis*. Only one study reported *Staphylococcus hominis* as the most common pathogen [64]. All groups showed a high resistance rate to ampicillin and clindamycin. A lower resistance was reported to imipenem, meropenem, and amikacin. Imipenem is one of the alternative drugs advised by the Saudi National Antimicrobial Therapy Guidelines [11]. There were high rates of sensitivity to aminoglycoside, tigecycline, gentamycin, and ciprofloxacin. Most of the isolated pathogens were reported to have been multidrug resistant [13,25,64]. Ciprofloxacin was suggested as the drug of choice for empirical therapy in 2001, and, still in 2022, another study reported high bacterial sensitivity to it, indicating that it is a safe choice for many clinicians. The rate of resistance to ciprofloxacin was reported as high as 34% [13,25].

### 3.3. UTI in Older Adults

UTIs associated with asymptomatic bacteriuria are a common challenge in older patients. This is due to the lack of localized genitourinary symptoms [70]. There were three papers in which older adults were specifically studied [32,33,65]. One of the studies reported that older patients were more prone to UTIs; 37.5% of urine cultures from older patients were positive for growth as compared to 24.34% and 32.29% in adults and children, respectively [33]. UTIs accounted for 14.6% of emergency department visits by older patients in 2018 and 11% in 2001 [32,65]. The prevalence of UTI was reported to be more common among female patients, but it tended to decrease with age. On the other hand, the prevalence among males tended to increase with age, especially among older men [33].

*E. coli*, *Klebsiella* species, and *Enterobacter* species accounted for most infections. There was a high resistance rate to ampicillin and trimoxazole commonly found among isolates [33,65]. UTI pathogens showed a higher resistance rate to antibiotics such as amoxicillin–clavulanic acid, ciprofloxacin, and nitrofurantoin among patients older than 65 years of age. Moreover, 47.2% of older patients experienced inappropriate antibiotic prescriptions, with inappropriate duration of treatment being the most common error (*p* < 0.05) [33]. Multidrug resistance was much more common among older people (50%) compared to other age groups (<20%), as was extended-spectrum β-lactamases (ESBL)-producing *E. coli* (8% in older people vs. 5% in other age groups) [32]. This high rate of resistance suggests the need to reevaluate and have an updated empiric therapy to combat the emergence of new resistant strains [32,33].

### 3.4. UTI in Cancer Patients

Two papers studied UTI prevalence in cancer patients. UTIs were the second most common infection after bloodstream infections [34,35]. The most common malignancies among the study groups were non-Hodgkin’s lymphoma and AML (acute myeloid leukemia), followed by colorectal cancer and other types of malignancies. Patients who undergo chemotherapy have a high risk of developing febrile neutropenia, characterized by a fever of more than 38 °C and a decreased neutrophil count (less than 500 cells/mm^3^), which is also associated with an increased risk for infection and mortality. Bacterial infections are among the major causes of mortality in neutropenic cancer patients [34,35]. 

In the past decades, there has been a shift in the prevalence of bacterial infections, from Gram-positive to Gram-negative being the most common. Studies conducted in the last decade reported *E. coli* and *Klebsiella* spp. as the most common Gram-negative bacteria pathogens [34,35]. Both pathogens were susceptible to imipenem-cilastatin, amikacin, piperacillin-tazobactam, and ceftriaxone. The ESBL production rate for *E. coli* and *Klebsiella* spp. were 38% and 22%, respectively [34,35].

### 3.5. UTI in Emergency Department Patients

UTIs are among the most common causes of emergency department (ED) visits, and the most common medical condition for which antibiotics are prescribed in many places around the world [71]. Four papers were found that included cases with UTI from emergency department [2,3,32,33]. UTI cases accounted for around 10% of total visits to the ED, with a higher prevalence of 13.3% in the month of January. Regarding age groups, 14.6% of older adults visited EDs due to UTI symptoms [3,32].

The male-to-female ratio was reported as 1:2 [3,32]. Women were most commonly affected by UTIs, except among older adults, where female patients were less than 50% of total older patients presenting with a UTI [3,33]. The average hospital stay of a patient with a UTI was around 3.5 days, and the longest stay was 23 days. Around 24% of patients required isolation, and 3% required admission to the ICU (intensive care unit).

*E. coli* was the most frequently isolated pathogen, followed by *Enterobacter* spp., *Klebsiella* spp., and *Acinetobacter* spp. *E. coli* showed resistance to ampicillin and co-trimoxazole and showed sensitivity to nitrofurantoin, followed by ciprofloxacin, amoxicillin–clavulanic acid, and cefazolin. ESBL-producing *E. coli* was detected in approximately 7% of the isolates [2,32,33].

Most of the patients (around 80%) were initially treated with broad-spectrum antibiotics. Cephalosporin and penicillin were the most common classes. Cefuroxime and norfloxacin were the most commonly prescribed antibiotics among adult and older adult patients, whereas in pediatric patients, the most common choice was amoxicillin–clavulanic acid followed by cefprozil [2,32,33].

UTIs account for a high economic cost for emergency departments. Only one study reported the economic impact of UTIs on EDs [32]. The cost of hospital treatment for a UTI in the ED of King Abdulaziz Medical City in 2018 ranged from USD 90.19 to USD 328.65 per patient, and an estimated yearly cost of USD 838,375. Another factor that contributes to the high cost is inappropriate prescription of antibiotics, which accounted for 47% of the overall cost (*p* < 0.05).

To some extent, we can say that there was an overestimation of UTIs among patients who visited EDs with UTI-like symptoms. This was confirmed with a 60% prevalence of positive cultures [3], and in some other cases only 30% of total ordered urine cultures from the ED were positive [32,33]. 

Another problem that was noted was the burden of readmissions. Around 10% to 15% of patients were found to be readmitted to the ED within 30 days after discharge, and the median time for readmission was around 7 days. Patients who required isolation were more likely to get readmitted. Readmission was more common in patients having underlying diseases [3]. Unplanned readmission is a huge burden on the hospital facilities, especially for emergency departments, as it increases the cost and overloads the setting. 

### 3.6. Inappropriate Antibiotic Prescriptions

The overuse and inappropriate use of antibiotics are two of the most important causes of antimicrobial resistance [72]. According to a report from the WHO, around 20% to 50% of antibiotics are inappropriately prescribed [2,6]. Only two papers identified this issue in KSA [2,32]. Inappropriate use of antibiotics includes dose errors, duration errors, frequency errors, and inappropriate selection of antibiotic class [2,32]. Around 47.3% of prescribed antibiotics in EDs were inappropriate. Pediatric patients were the most vulnerable group (*p* < 0.001); 57.8% were prescribed inappropriate antibiotics as compared to a rate of 37.8% among adult patients. Pediatric patients were more prone to errors such as dosage (*p* < 0.001) and duration errors (*p* < 0.001), while adult patients were more prone to errors such as inappropriate selection of antibiotic class (*p* < 0.001). However, neither study reported a significant association between age and frequency of selection error (*p* = 0.74) [2,32].

Inappropriate antimicrobial prescription for a UTI is more common in the ED as EDs often prescribe antibiotics. Using a lower concentration of antibiotic or for a shorter duration will cause antibacterial resistance, and overuse will result in toxicity, an increased antimicrobial resistance rate, or other side effects. Inappropriate prescription is also associated with several clinical manifestations such as allergies, gastrointestinal disturbances, renal/liver distress, etc. Assessing the weight of an infant before prescribing a dosage of any antibiotic is quite important. Studies show that even though the weight of the infant was measured, there were still errors in dosage calculation, with overdose being the most common calculation error [2,32].

Inappropriate selection of antibiotics is related to an increased antibiotic resistance rate, along with several complications and an increased chance for readmission. It will also affect the healthcare system as it will increase the cost of treatment and make the battle against antimicrobial resistance more difficult. There was an interesting correlation between diagnostic tests, prescription inappropriateness, and cost. Patients who had undergone diagnostic tests had a higher incidence of being prescribed expensive antibiotics. On the other hand, patients who did not undergo diagnostic tests initially had a lower cost, but they were inappropriately prescribed antibiotics, causing many later complications [2,32].

Cephalosporin was 3.31 times more likely to be inappropriately prescribed to adult patients, compared to penicillin prescriptions. On the other hand, penicillin was more likely to be inappropriately prescribed to pediatric patients (33.6%) compared to other antibiotics (*p* < 0.05). Cephalosporin was the most inappropriately prescribed antibiotic (more than 90%). Most of the patients who were administered or prescribed antibiotics were not screened for antibiotic allergy; only 7% of patients were screened for antibiotic allergy prior to its administration or prescription.

### 3.7. Prevalence of ESBL

Thirteen articles were found that mentioned and tested ESBL-producing uropathogens. They were detected in approximately 2% [39], 7% [32], 8% [26], 9% [16], 11% [41], 12% [17], 15% [13], 17% [64], 20% [38], 45% [37], 33% [68], 43.5% [23], and 44% [51] of urinary specimens. ESBL species have been a major concern for healthcare systems all around the world since 2000 when their outbreak started [16,68].

ESBL-producing *E. coli* are much more prevalent among women than men. ESBL-producing *E. coli* were resistant to gentamicin, ceftazidime, amoxicillin–clavulanic acid, cefotaxime, levofloxacin, and ciprofloxacin, and they showed sensitivity towards carbapenems, amikacin, and piperacillin/tazobactam [18,19,21,23,24,30]. ESBL-producing *Klebsiella* spp. showed resistance to piperacillin, cefotaxime, and ceftazidime and sensitivity towards piperacillin-tazobactam, amikacin, and carbapenems [14,18,41].

### 3.8. UTI in Children

Twelve papers reported UTIs in children (0–12 years old) [2,15,17,19,20,21,22,32,33,50,59,62]. UTIs account for 4% to 15% of total ED visits by pediatric patients [2,32]. UTIs and related complications account for around 5% of ED visits by children, compared to 15% of ED visits by other age groups. UTIs in children are common but difficult to diagnose due to their ambiguous nature. On the other hand, they have a very good prognosis [15,50]. Girls are at higher risk for infection. Up to the age of 6 years, the incidence rate is similar for both sexes, and this could be due to hygiene or other causes, but later the incidence among boys starts to decrease [15,21,50]. Circumcision is significantly associated with the development of UTIs among young boys. Uncircumcised boys are more prone to UTI than circumcised boys [20].

Some studies performed renal ultrasound on pediatric patients with a confirmed UTI. The most commonly reported abnormalities were hydronephrosis, uretric dilatation, dilatation of the renal calices and dilatation of the collecting system. Patients with abnormal ultrasound findings were associated with repeated episodes of UTI as compared to patients with normal findings who had only a single episode (*p* < 0.05). Moreover, abnormal ultrasound findings were significantly more common in non-*E. coli* UTIs [20,21,22]. 

As UTIs are common among children, they can be severe and have serious complications. A common complication is vesicoureteral reflux. Renal ultrasound is a good method to detect this complication [20,21,22]. Vesicoureteral reflux can be found in around 30% of children affected by UTI and bilateral reflux was very common as well. Patients who had vesicoureteral reflux had a high risk of developing pyelonephritis and scarring. In addition, renal parenchymal scarring was reported as a common complication of delayed treatment [20,21,22].

*E. coli* was reported as the most common isolated organism, followed by *Klebsiella* spp., *E. faecalis*, and methicillin-resistant *Staphylococcus epidermidis* (MRSE) [2,15,17,19,20,21,22,32,33,50,59,62]. Non-*E. coli* infections were found to be more common in young boys (*p* < 0.0001), but overall, young girls were much more prone to UTI [15].

*E. coli* showed resistance against ampicillin, cephalothin, co-trimoxazole, and cefoxitin and was sensitive to cefotaxime, ceftazidime, colistin, levofloxacin, and tigecycline [2,15,17,19,20,21,22,32,33,50,59,62].

*Klebsiella* spp. were highly resistant against ampicillin, cefoxitin, cefuroxime, cephalothin, and tobramycin. It showed less resistance to co-trimoxazole. *Klebsiella* spp. were highly sensitive to amikacin, aztreonam, cefepime, ciprofloxacin, gentamicin, imipenem, and levofloxacin, followed by meropenem, nitrofurantoin, norfloxacin, ceftriaxone, tazocin, and amoxicillin/clavulanic acid [17,18,23].

*E. faecalis* was not a commonly isolated pathogen. It was found to be highly sensitive to cephalothin, ciprofloxacin, linezolid, nitrofurantoin, teicoplanin, and vancomycin, but it was highly resistant to cefepime and tetracycline, followed by clindamycin and erythromycin, cefoxitin, gentamicin, and ampicillin [17].

Inappropriate antibiotic prescriptions were most common among pediatric patients, accounting for 51.3% of all prescriptions [16]. Some recent studies suggest the use of third-generation cephalosporin as a broad spectrum for community-acquired UTI because the majority of uropathogens were sensitive to it (96%) [17,19].

### 3.9. UTI in Adults

Urinary tract infections are among the most common infections worldwide, and they affect people from all age groups, especially women. The WHO has reported that an estimated 50% of women experience a UTI at some point in their lives [18]. We found 18 papers that studied UTIs in adults, especially in women, as they experienced more infections than men [2,13,18,24,26,27,28,29,30,32,33,36,40,43,49,55,58,60]. Adult patients were the most represented in all the studies. In KSA, UTIs were reported to be the second leading cause of infection (predominantly in women) at EDs. Adult patients account for the most visits to an ED presenting with symptoms of UTI [2]. The vast majority of healthy adults who have a UTI will present with mild symptoms and can recover from the infection with a simple medication plan. The problem is reoccurrence, and using ineffective antibiotics will lead to an increased rate of resistance and urinary tract-related complications. On a large scale, many of the studies reported female patients as more likely to present with recurring UTIs [2,36,55].

The most commonly isolated organisms were *E. coli*, followed by *Klebsiella* spp., *P. aeruginosa S. agalactiae*, *E. faecalis*, *Streptococcus group B and D*, methicillin-resistant *Staphylococcus Aureus* (MRSA), and vancomycin-resistant *E. faecalis* (VRE). Some studies even reported a significantly higher prevalence of UTI caused by *E. coli* among women (*p* < 0.05) [2,13,18,24,26,27,28,29,30,32,33,36,40,43,49,55,58,60]. 

*E. coli* isolates showed a high resistance rate to ampicillin, co-trimoxazole, mezlocillin, piperacillin, trimethoprim, ciprofloxacin, and to cefazolin. On the other hand, they showed a high sensitivity rate to amikacin, ertapenem, nitrofurantoin ciprofloxacin, levofloxacin, moxifloxacin imipenem, meropenem, norfloxacin, and piperacillin/tazobactam [2,13,18,24,26,27,28,29,30,32,33,36,40,43,49,55,58,60]. 

*Klebsiella* spp. was the second most common pathogen, and it showed the highest resistance rate to ampicillin co-trimoxazole, followed by cefuroxime, aztreonam, cefepime, ceftriaxone, cefuroxime, cephalothin, ceftazidime, and amoxicillin. Additionally, *Klebsiella* spp. showed a high susceptibility rate to meropenem, imipenem, colistin, ertapenem, amikacin, and levofloxacin. The antimicrobial resistance patterns of other less common pathogens were similar to *E. coli* and *Klebsiella* spp. Ertapenem and imipenem are the first line of defense against ESBL-producing *Enterobacter* according to the Saudi National Antimicrobial Therapy Guidelines [11].

### 3.10. UTI in Pregnant Women

UTIs are very often encountered among women, especially pregnant women [73]. Three papers reported on UTIs in pregnant women [13,44,52]. The first one was published in 1991 [44], a second in 2013 [52], and the most recent in 2018 [13].

In the 1991 study [44], the prevalence of UTI among pregnant women was 16%. However, in the study that was conducted in the last decade, the prevalence of UTI was as high as 20% [52]. In both studies, around half of the patients who tested positive for UTI were asymptomatic. Patients with symptomatic UTI were significantly more likely to have a premature birth as compared to the asymptomatic group [44]. Asymptomatic UTIs can spread to the upper renal system and cause pyelonephritis, which is a major cause of septic shock among pregnant women [44,52].

UTIs during pregnancy were reported to be a common problem and they were commonly associated with several disorders [44,52]. Pre-eclampsia (a high blood pressure disorder that can occur during pregnancy) tended to be more common among patients with UTI. There was also a significant relationship between the presence of bacteriuria and development of conditions such as anemia and hypertension [44]. The incidence rate was similar between symptomatic and asymptomatic women [44,52]. 

The most common causative agents of UTI among pregnant women were *E. coli, Staphylococcus*, and *Candida* species, which is an indication of fecal contamination and poor personal hygiene [44,52]. 

Amoxicillin, cefoxitin, ceftazidime, norfloxacin, penicillin, and fusidic acid were the most useful antibiotics for treating UTIs as they showed the least resistance frequency. On the other hand, ampicillin and tetracycline had the highest resistance [13,44,52].

### 3.11. Catheter-Associated UTIs

Catheter-associated urinary tract infections (CAUTIs) are among the most frequent hospital-acquired infections. There were around eleven publications on CAUTIs [45,47,48,54,56,57,61,62,66,67,74]. CAUTIs are the most common nosocomial infections in KSA, where they account for 7% [54], 20% [61] 22% [66], 24.4% [45], 28.5% [57], and 42% [48] of hospital-acquired infections. There are several risk factors for the occurrence of CAUTI among ICU patients, such as prolonged hospitalization, prolonged catheter usage, poor aseptic technique, inappropriate use of antibiotics, gender, extremes of age, immunosuppressive drugs, etc. Sixty to seventy percent of CAUTIs can be prevented by proper infection control and by implementing preventive bundles [67]. Another risk factor related not only to CAUTIs but also to nosocomial infections in general is that they are caused by pathogens with very strong resistance rates and are a huge challenge to treat, especially for immunocompromised patients [45,54,61].

CAUTIs were more common in older patients (60 to 80 years old) [66,74]. The main causative pathogens were *Klebsiella* spp., followed by *E. coli* and *Proteus*, with a high sensitivity to amikacin, gentamicin, and carbapenem [54,66]. However, most of the articles did not report the main pathogens or their antimicrobial resistance patterns.

Some studies reported on interventions over a long period of time and tried to reduce the rate of nosocomial infections. Before the intervention, the rate of CAUTIs among patients admitted to ICU was 2.3 per 1000 days. After initial screening of the catheter and patients who were catheterized in ICU and with proper documentation and discouragement of routine replacement, the infection rate dropped to 1.9 per 1000 days in a short period [45]. In another intervention that lasted 2 years, the CAUTI rate dropped from 3.5 per 1000 days before the intervention to 2.9 per 1000 days after 1 year, to 2.2 in the beginning of the second year, and to 1.7 at the end of the last year [56]. In a 7-year surveillance and intervention, the CAUTI rate dropped from 6.75 per 1000 days to 3.41 per 1000 days [48]. The CAUTI rate dropped significantly after the intervention, but there is another noticeable fact. The early 2000s study [48] reported an incidence rate of around 10 per 1000 days, and the most recent study reported an incidence rate as low as 0.3 per 1000 days [56]. This is an indication that the standard of healthcare facilities and the adherence to infection control guidelines has improved.

### 3.12. Inpatient vs. Outpatient Studies

Most of the studies were retrospective in nature and took into consideration only cases with positive urine cultures; they did not categorize inpatients or outpatients or specify which clinic was visited. Some studies focused only on the patients who visited EDs, as mentioned in Section 3.5 [2,3,32,33]. However, some studies focused only on inpatients and mostly on CAUTIs; as mentioned in Section 3.11, they did not report causative pathogens or their antimicrobial resistance patterns [31,45,48,56,57,61,62]. Throughout this review, *E.coli* was found to be the most common uropathogen, except in some studies that reported inpatient data where *Klebsiella* spp. was the main causative pathogen [35,54,66]. There was a higher MDR rate among inpatient samples [54]. Additionally, when the hospital stay was prolonged (>8 days), the risk for acquiring a UTI was significantly higher [45]. Two studies specifically reported on outpatients from nephrology clinics, and *E.coli* was reported as the main causative pathogen, but neither of those two studies reported the antimicrobial resistance pattern for those uropathogens [14,50].

## 4. Discussion

There were 58 publications published in KSA about UTIs, UTI prevalence, and the main UTI pathogens. UTIs were much more common among women than men. However, the prevalence was approximately equal between the sexes among inpatient CAUTI cases and among children under the age of 5 years [4,63,75]. Among the reasons why UTIs are more frequent among women is the anatomical structure of their urogenital system. Children under 5 years have a similar infection rate for hygiene reasons, but, as they mature, they tend to become more aware of hygiene and eventually decrease the risk. Another factor in the incidence of UTI among children is circumcision as uncircumcised boys tend to have a higher prevalence of UTI [9,21].

In our review, the *Enterobacteriaceae* family was the main cause for UTI. *E. coli* and *Klebsiella* spp. were the most common isolated organisms, and in some studies, they were the only reported organisms. They tended to have a strong antimicrobial resistance pattern; those isolated from inpatients were more likely to present with MDR and a high ESBL production rate [51,64,76]. Another major problem contributing to the high resistance rate seems to be inappropriate antibiotic prescription. There was a high prevalence of inappropriate antibiotic prescriptions, especially in emergency rooms, which affected mostly children and older adult patients. This might be among the reasons why patients with UTI are admitted to ED very frequently. It was also reported that there was a high readmission rate among patients with inappropriate antimicrobial prescriptions. This issue was addressed only by two papers, and they revealed major inappropriateness and recommended more focus be given to this issue [2,32].

Hospital-acquired UTIs, especially CAUTIs, are a major challenge, and investigation, the measures taken, and incentives given seem to have had a good effect because these infections have significantly decreased in the last two decades [56,66,77]. The CAUTI incidence was similar between males and females; this could be due to immunosuppression, catheter usage, and/or other factors related to nosocomial infections [56].

Women in general are at high risk for developing UTIs, and pregnancy tends to increase the risk. The presence of a UTI seems to be related to other conditions during pregnancy, such as anemia, hypertension and even premature birth [69,73]. A similar risk of UTI was found among patients with diabetes; infection tended to be associated with uncontrolled diabetes [14,31,46]. These associations might be due to immunosuppression that pregnant women and diabetic patients experience.

All the included studies had adequate sample sizes. All studies enrolled more than 100 cases; only five had a sample of less than 100, and a few had more than 1000 cases. Many of the papers assessed other medical conditions (e.g., pregnancy, cancer, diabetes, and intensive care unit) and their correlation with UTI prevalence.

Most of the papers were descriptive papers and did not have any significant statistical analysis. Moreover, apart from a few papers, most studies were retrospective, included only UTI-positive samples, and were not compared to a control group to assess the risk factors for developing a UTI, and resulted in a 100% prevalence rate [12,13,15,16,18,19,20,21,24,25,26,27,28,29,30,32,36,37,41,43,50,51,52,55,58,59,60,67]. Most of the publications were retrospective and lacked data on patients’ course of treatment and the prevalence of UTI reoccurrence and complications. Additionally, not all regions were equally represented. As mentioned above, some regions were continuously studied, while some others were represented in only one or two studies.

Antimicrobial resistance is growing at a concerning rate. A continuous, more extensive, and inclusive study is needed to understand the full antimicrobial sensitivity pattern both locally and nationally in KSA and to plan an updated treatment regimen.

Improper antibiotic prescription is another factor that leads to complicated UTI and an increased rate of antimicrobial resistance, but, unfortunately, only a few papers highlighted this issue. The nature of most studies was retrospective, and there was no patient follow-up until the end of the treatment and recovery from the infection. Thus, data were limited on the appropriateness of the treatments. Moreover, the data of prescribed empirical therapy and its appropriateness for the microbiology report were not reported. Future studies should focus on these matters.

This review paper was the outcome of a comprehensive search of multiple databases with specific keywords and careful examination of the titles and manuscripts of found publications. Therefore, it is unlikely that we missed any relevant English studies. However, any publication in Arabic only or nonpublished data on UTIs in KSA may have been missed. Additionally, some papers may have been excluded due to a lack of clearly specified samples/populations.

## 5. Conclusions

UTIs remain a major challenge for the healthcare system. Women and patients with underlying conditions tend to be at a higher risk, and *E. coli* remains the major causative pathogen. The current major concern is the rising antimicrobial resistance rate which requires rigorous scientific inquiry into antimicrobial resistance. Large epidemiological studies at the regional and national levels are needed to battle the rapid emergence of antimicrobial resistance. On the other hand, prospective and longitudinal studies are needed to assess the effectiveness of initially prescribed antibiotics. The data that can be obtained from these future studies will be valuable in developing new guidelines for the use of empiric therapies and controlling the increased rate of antimicrobial resistance.

## Figures and Tables

**Table 1:** General description of included urinary tract infection (UTI) studies (n = 58).

#	First Author, Year	Design	Location	Sample Size	Patient Type	Age Range (Years)/Sex (M/F)	UTI Prevalence	Most Common Pathogens	Antibiotic Resistance	Significant Correlates of UTI	Additional Comments
1	Ahmed T. Eltahawy, 1988 [43]	Prospective	Jeddah	575	Patients presenting with urinary tract infection (UTI)	All ages (M/F)	100%	*E. coli*, *Klebsiella* spp., *Enterobacter* spp., and *Pseudomonas* spp.	Sulfamethoxazole (78%), ampicillin (64%), tetracycline (62%) and carbenicillin (64%).	Not mentioned	Not mentioned
2	Ali Magzoub El-Bashier, 1991 [40]	Retrospective	Qatif	13,193	Patients with suspected UTI	All ages (M/F)	7.6%	*E. coli* and *Klebsiella* spp., *Enterobacter* spp.	Ampicillin (70%), piperacillin (58%)	Not mentioned	Norfloxacin be considered an empirical therapy.
3	Hassan Abduljabbar, 1991 [44]	Prospective	Jeddah	2642	Pregnant patients presenting with suspected UTI	All ages (F)	15.8%	*E. coli* and *Klebsiella* spp.	Not mentioned	Symptomatic group had a higher risk for premature birth (*p* < 0.05).	Hypertension and anemia in pregnancy were more common in women with bacteriuria.
4	D H Akbar, 2001 [25]	Retrospective	Jeddah	182	Patients presenting with UTI	All ages (M/F)	100%	*E. coli* and *Pseudomonas* spp.	Not mentioned	Diabetes	Aminoglycoside and ciprofloxacin can be used empirically.
5	Abdulrahman A. Kader, 2001 [26]	Retrospective	Al-Khobar	2394	Patients presenting with UTI	All ages (M/F)	100%	*E. coli*, *Klebsiella* spp. and *Pseudomonas* spp.	Trimethoprim (47%) and amoxycillin (62%).	Not mentioned	Not mentioned
6	Alia Abdulrahim Al-Ibrahim, 2002 [22]	Retrospective	Riyadh	82	Patients presenting with UTI	0–5 (M/F)	100%	*E. coli* and *Klebsiella* spp.	Not mentioned	Half of the patients in the study had bilateral reflux.	Hypertension scar formation and renal impairment were not detected.
7	Saeed M. Al-Asmar, 2004 [31]	Case-control	Riyadh	824	Inpatients	All ages (M/F)	25%	Not mentioned	Not mentioned	UTIs account for one-third of nosocomial infections.	Not mentioned
8	Abdulrahman Abdulla Kader, 2004 [39]	Not mentioned	AL Khobar	11,659	Patients with suspected UTI	All ages (M/F)	17.5%	*E. coli*, *Klebsiella* spp., *Pseudomonas* spp., *Proteus* spp.	Amoxicillin (61%) and trimethoprim (47%)	Not mentioned	There was noticeable antibiotic resistance.
9	Abdulrahman Abdulla Kader, 2005 [16]	Retrospective	Dammam	2302	Patients admitted with UTI	All ages (M/F)	100%	*E. coli*, *Klebsiella* spp., *Enterobacter* spp.	Cefepime (88.5%), ciprofloxacin (86%) and gentamicin (77.5%)	Not mentioned	There is a high presence of *ESBL* producers in uropathogens among inpatients and outpatients.
10	Abdulrahman Abdulla Kader, 2005 [41]	Retrospective	Alkhobar and Dammam	2455	Patients presenting with UTI	All ages (M/F)	100%	*E. coli* and *Klebsiella* spp.	ESBL-production (11%), cefepime (78%), ciprofloxacin (45%)	Not mentioned	It is important to apply measures to restrict the spread of ESBL infections.
11	Hanan H. Balkhy, 2006 [45]	Cross-sectional	Riyadh	562	Inpatients	All ages (M/F)	38 (8%)	Not mentioned	Not mentioned	Hospital stay exceeding 8 days	Urinary catheters were reported as an important source of infection.
12	Abdulla A Al-Harthi, 2008 [23]	Retrospective	Aseer	464	Patients presenting with UTI	0–12 (M/F)	100%	*E. coli*, *Klebsiella* spp. and *Pseudomonas* spp.	Not mentioned	Not mentioned	Ceftriaxone, imipenem, and azactam are appropriate for initial empirical therapy.
13	Layla Alshamsan, 2009 [21]	Retrospective	Riyadh	130	Patients presenting with UTI	0–12 (M/F)	100%	*E. coli*, and *Klebsiella* spp.	Not mentioned	Not mentioned	Renal ultrasound has little value in the management of children with UTI.
14	Sameera M. Al Johani, 2010 [24]	Retrospective	Riyadh	2792	Patients presenting with UTI	All ages (M/F)	100%	*Acinetobacter baumannii*, *Pseudomonas* spp., *E. coli*, *Klebsiella* spp.	Amikacin (94%), imipenem (90%), meropenem (90%) and ciprofloxacin (90%).	Not mentioned	Antimicrobial resistance is an emerging problem in the intensive care unit (ICU).
15	Khalid A Al-Rubeaan, 2013 [46]	Cross-sectional	Riyadh	1000	Diabetic patients	All ages (M/F)	25.3%	Not mentioned	Not mentioned	The incidence of UTI in both type 1 and 2 diabetics was similar.	The body mass index, hypertension, microalbuminuria and insulin therapy were significantly higher in patients with UTI.
16	DA Abdulmutalib, 2013 [47]	Prospective	Taif	Not clearly specified	Inpatients	All ages (M/F)	Not clearly specified	*E. coli* and *Pseudomonas* spp.	Not mentioned	Not mentioned	Catheter associated urinary tract infections (CAUTIs) declined from 3.5 to 2.2 per 1000 catheter-days in 2013.
17	Jaffar A. Al Tawfiq, 2013 [48]	Prospective	Dhahran	Not clearly specified	Inpatients	All ages (M/F)	Not clearly specified	Not mentioned	Not mentioned	CAUTI was the most common nosocomial infection.	The use of preventive bundles was effective in decreasing the infection cases.
18	Md. Afzal Hossain, 2013 [49]	Cross-sectional	Riyadh	510	Patients presenting with UTI	All ages (M/F)	100%	*E. coli*, *Klebsiella* spp. and *Pseudomonas* spp.	Ampicillin (84%), cephalothin (75%), and co-trimoxazole (62%).	Not mentioned	Ciprofloxacin resistance was also closely associated with multidrug resistance.
19	Abdulla A. Alharthi, 2014 [50]	Cross-sectional	Taif	1000	Outpatients	3–6 (M/F)	5%	*E. coli* and *Enterococci*	Not mentioned	25% of the screened children had urinary abnormalities.	Pyuria were evident in 5% of cases and hematuria in 2.5%.
20	Mansoor Sirkhazi, 2014 [34]	Retrospective	Dammam	106	Neutropenic cancer patients	All ages (M/F)	29.71%	*E. coli*, *Klebsiella* spp., *Staphylococcus aureus*	Not mentioned	Cancer patients with febrile neutropenia	The use of initial antibiotic therapy in febrile neutropenic episodes should be based on local bacterial spectrum.
21	Mohamed H Al-Agamy, 2014 [38]	Retrospective	Riyadh	152	Patients presenting with UTI	All ages (M/F)	100%	*E. coli*	ESBL-production 20%	Not mentioned	Not mentioned
22	Menyfah Alanazi, 2015 [2]	Cross-sectional	Riyadh	5752	Patients presenting in emergency department	All ages and (M/F)	24.9%	*E. coli* and *Klebsiella* spp.	Penicillin and cephalosporin showed the highest resistance rates, but data was not provided.	Not mentioned	Penicillin and cephalosporin were the most common wrongly prescribed antibiotics.
23	TA El-Kersh, 2015 [51]	Not mentioned	Khamis Mushayt	269	Patients presenting with UTI	All ages (M/F)	100%	*E. coli* and *Klebsiella* spp.	Co-trimoxazole (53%) and nitrofurantoin (25%)	Not mentioned	Not mentioned
24	Wallaa A. Garout, 2015 [20]	Retrospective	Riyadh	153	Patients presenting with UTI	0–5 (M/F)	100%	*E. coli*, followed by *Klebsiella* spp.	Not mentioned	A single episode of UTI signified normal urological anatomy.	Urological anomalies were found in 28.1% of the overall study population.
25	Hani S. Faidah, 2015 [52]	Prospective	Mecca	200	Pregnant patients presenting with UTI	18 to 45 years (F)	100%	*E. coli* and *Klebsiella* spp.	Ampicillin (55%) and tetracycline (34%)	UTI is very common among pregnant women in Mecca.	Not mentioned
26	Sulaiman Ali Al Yousef, 2016 [53]	Cross-sectional	Hafr Al Batin	908	Patients with suspected UTI	All ages (M/F)	75%	*E. coli* and *Klebsiella* spp.	Ampicillin (90%), mezlocillin (88%) and co-trimoxazole (66%)	Not mentioned	High resistance to commonly prescribed empirical therapy was observed.
27	Mohamed S. Kabbani, 2016 [54]	Retrospective	Riyadh	413	Inpatients	Children who underwent cardiac surgery (M/F)	7%	*Klebsiella* spp. and *E. coli*	33% of the pathogens were multidrug resistant.	Long duration of catheters	Resistant Gram-negative bacteria are an emerging concern in ICUs.
28	Samiah HS Al-Mijalli, 2017 [55]	Prospective	Riyadh	116	Patients presenting with UTI	All ages (M/F)	100%	*E. coli*, *Klebsiella* spp. and *Pseudomonas* spp.	Imipenem (98%), meropenem (98%) and ampicillin (95%)	Not mentioned	Pathogens were susceptible to meropenem, imipenem, colistin, and ertapenem.
29	Fahad M. Al-Hameed, 2018 [56]	Prospective	Jeddah	Not clearly specified	Inpatients	All ages (M/F)	Not clearly specified	Not mentioned	Not mentioned	Not mentioned	The monthly rates of CAUTI significantly declined after the enforcement of preventive strategies.
30	Eiman Gaid, 2018 [57]	Prospective	Different hospitals from different regions	6178	Inpatients	All ages (M/F)	28.4%	Not mentioned	Not mentioned	Not mentioned	CAUTI occurred from 2.3 to 4.4 per 1000 device-days.
31	Osama Al Wutayd, 2018 [58]	Cross-sectional	Buraidah	418	Patients presenting with UTI	All ages (M/F)	100%	*E. coli*, *Klebsiella* spp., *Proteus mirabilis* and *Pseudomonas* spp.	Ampicillin (89%), oxacillin (75%), and piperacillin (85%).	Not mentioned	There is a high multidrug resistance rate.
32	Bander Balkhi, 2018 [13]	Retrospective	Riyadh	1918	Patients presenting with UTI	All ages and (M/F)	100%	*E. coli*, *Klebsiella* spp. and *Pseudomonas* spp.	Co-trimoxazole (47%) followed by ciprofloxacin (34%)	Diabetes and pregnancy	The development of regional and national UTI guidelines is recommended.
33	Menyfah Q Alanazi, 2018 [32]	Retrospective	Riyadh	1449	Patients visiting emergency department	All ages (M/F)	9.9%	*E. coli*	Not mentioned	Not mentioned	There is a significant level of inappropriate use of antibiotics in the treatment of UTIs in the emergency department.
34	Abdulaziz Alqasim, 2018 [37]	Retrospective	Riyadh	100	Patients presenting with UTI	All ages (M/F)	100%	*E. coli*	ESBL production (67%) ampicillin (92%) and to amoxicillin–clavulanic acid (55%).	Not mentioned	67% of ESBL were multidrug resistant (MDR).
35	Menyfah Q. Alanazi, 2018 [33]	Retrospective	Riyadh	565	Patients admitted with UTI in emergency department	All ages (M/F)	100%	*E. coli*	Ampicillin (35%) and co-trimoxazole (43%)	Not mentioned	Higher resistance rate was noticed in young patients <12 years.
36	Salem K. Albalawi, 2018 [59]	Not mentioned	Tabuk	210	Patients presenting with UTI	0–12 (M/F)	100%	*E. coli* and *Klebsiella* spp.	Ampicillin (87%) and cotrimoxazole (81%) was observed.	Not mentioned	For *E. coli* the lowest resistance rate was for nitrofurantoin.
37	Abdulaziz Alamri, 2018 [27]	Retrospective	Aseer	49,779	Patients presenting with UTI	All ages (M/F)	100%	*E. coli* and *Klebsiella* spp.	Cephalothin (90%), nalidixic acid (87 %), and ampicillin (82%).	Not mentioned	Not mentioned
38	Ibrahim Taher, 2019 [60]	Retrospective	Aljouf	415	Patients presenting with UTI	All ages (M/F)	100%	*E. coli*, *Klebsiella* spp., and *Pseudomonas* spp.	Ampicillin (84%) and co-trimoxazole (53%).	Not mentioned	There is a high incidence of MDR strains.
39	Sulaiman I. A. Alsohaim, 2019 [29]	Retrospective	Buraidah	379	Patients presenting with UTI	All ages (M/F)	100%	*E. coli*, *Klebsiella* spp., and *Pseudomonas* spp.	Cefoxitin (71%) and gentamicin (48%).	Not mentioned	There was significant negative relationship between antimicrobial prescribing and resistance.
40	Syed Suhail Ahmed, 2019 [30]	Retrospective	Buraidah, Qassim	273	Patients presenting with UTI	All ages (M/F)	100%	*E. coli*, *Klebsiella* spp., and *Proteus* spp.	Ampicillin (88%), piperacillin (72%), clindamycin (66%) and amoxicillin/clavulanic acid (66%).	Not mentioned	There is high incidence of multidrug-resistant strains.
41	Hameed T, 2019 [15]	Retrospective	Riyadh	202	Patients admitted with UTI	Pediatric patients 0–14 (M/F)	100%	*E. coli* followed *by Klebsiella* spp., *Pseudomonas* spp. and *Enterococcus*	Ampicillin (68%) and co-trimoxazole (54%).	Not mentioned	For children with a community-acquired UTI, a third-generation cephalosporin is a safe choice.
42	Majid M. Alshamrani, 2019 [61]	Not mentioned	Different hospitals from different regions	1666	Inpatients	All ages (M/F)	6.8%	Not mentioned	Not mentioned	Not mentioned	Hospital-acquired UTIs accounted for 20% of all nosocomial infections.
43	Nehad J. Ahmed, 2021 [62]	Retrospective	Alkharj	7703	Inpatients	All ages (M/F)	0.5%	Not mentioned	Not mentioned	The rate of overall healthcare-associated infections was low.	The compliance rate to preventive measures was high.
44	Abdulrahman S Bazaid, 2021 [36]	Retrospective	Ha’il	428	Patients presenting with UTI	All ages (M/F)	100%	*E. coli*, *Klebsiella* spp., and *Staphylococcus aureus*	Piperacillin (45%) and co-trimoxazole (40%).	Not mentioned	Carbapenem and linezolid can be considered first therapeutic choices.
45	Yaser Saleh Bamshmous, 2021 [19]	Retrospective	Jeddah	278	Patients presenting with UTI	0–16 (M/F)	100%	*Staphylococcus*, followed by *Klebsiella* spp.	Data was not provided	Not mentioned	Not mentioned
46	Mohammed Abdullah Alzahrani, 2021 [17]	Retrospective	Al-Baha	118	Patients admitted with UTI	Pediatric patients 0–14 (M/F)	100%	*E. coli*, ESBL *E. coli*, *Klebsiella* spp., *Enterococcus faecalis*	Ampicillin (94%), cephalothin (92%), and cefoxitin (76%).	Not mentioned	Antibiotic resistance can be reduced compliance to guidelines.
47	Mohammed Yahia Alasmary, 2021 [18]	Retrospective	Najran	136	Patients presenting with UTI	All ages (M/F)	100%	*E. coli*, and *Klebsiella* spp.	Ampicillin (63%) and cephazolin (60%).	Not mentioned	The patients with UTIs in the Najran region of KSA are at a high risk of antibiotic resistance.
48	Mariam Alrasheedy, 2021 [63]	Cross-sectional	Ministry of Health hospitals in KSA	1083	Patients with suspected UTI	0–10 year sold (M/F)	25.8%	*E. coli*, *Proteus*, *Klebsiella* spp., *Enterococcus*, *Citrobacter*	Not mentioned	Not mentioned	Nearly a sixth of children could develop severe/complicated UTI.
49	Abdulaziz Alamri, 2021 [12]	Retrospective	Abha	1506	Patients presenting with UTI	All ages and (M/F)	100%	*E. coli* and *Klebsiella* spp.	Ampicillin (91%) and cephalothin (93%).	Not mentioned	Fosfomycin, cefoxitin, nitrofurantoin, are recommended as first-line treatment.
50	Lina Almaiman, 2021 [14]	Retrospective	Buraidah	754	Outpatients of nephrology clinic	14–95 (M/F)	21.8%	*E. coli* and *Klebsiella* spp.	Not mentioned	Chronic kidney disease (CKD).	The management of comorbidities could help to control the progression of CKD to the late stages.
51	Saad Alghamdi, 2021 [35]	Retrospective	Mecca	678	Cancer patients	All ages (M/F)	44%	*Klebsiella* spp., *E. coli* and *Pseudomonas* spp.	Fluoroquinolones (50%), cephalosporin (50%) and carbapenems (50%).	Not mentioned.	Enhanced antibiotic resistance was found by Gram-negative bacilli.
52	Kawther Aabed, 2021 [64]	Prospective	Riyadh	113	Patients presenting with UTI	All ages (M/F)	17.5%	*E. coli*	Norfloxacin (80%), amoxicillin (70%) ampicillin (70%) and co-trimoxazole (55%).	Not mentioned	Not mentioned
53	Samiah Hamad S Al-Mijalli, 2022 [65]	Cross-sectional	Riyadh	100	Type 2 diabetes patients	All ages (M/F)	22%	*Streptococcus*, and *Pseudomonas* spp.	Tigecycline (88%), gentamycin (84%) and nitrofurantoin (78%).	Diabetic patients are prone to a wide range of pathogens.	Tigecycline can be used as empirical treatment.
54	Bader S Alotaibi, 2022 [28]	Retrospective	Aljouf	1334	Patients presenting with UTI	All ages (M/F)	100%	*E. coli*, *Klebsiella* spp., and *E. faecalis*	Carbapenems (37%) and) to meropenem (34%).	Not mentioned	MDR gram-negative bacteria dominate the pathogenic spectrum of UTI.
55	Mohd Saleem, 2022 [66]	Cross-sectional	Ha’il	1078	Inpatients	Older than 18 years (M/F)	6.5%	*Klebsiella* spp.	Mupirocin (80%), tigecycline (80%) and ceftriaxone (47%).	Not mentioned	It is recommended that a smaller-size catheter be used to provide better drainage.
56	Yvonne S. Aldecoa, 2022 [67]	Prospective	Different hospitals	919,615 patient-days	Patients with a urinary catheter	All ages (M/F)	965 cases	Not mentioned	Not mentioned	Not mentioned	CAUTI rate was 1.68 per 1000 urinary-catheter days.
57	Sarah Alrashid, 2022 [3]	Retrospective	Riyadh	315	Patients presenting with UTI	All ages (M/F)	100%	*E. coli*, *Klebsiella* spp., and *Pseudomonas* spp.	Ampicillin (58%) and co-trimoxazole (42%).	Not mentioned	High resistance was found against antibiotics used as empirical therapy.
58	Adil Abalkhail, 2022 [68]	Randomized experimental study	Riyadh	2250	Patients presenting with UTI	All ages (M/F)	100%	*E. coli*	Ampicillin (100%) cephalosporins (90%) ESBL-production (33%).	Not mentioned	High resistance was found against antibiotics used as empirical therapy against ESBL.

CAUTI: catheter-associated urinary tract infection; CKD: chronic kidney disease; ESBL: extended-spectrum β-lactamases; *E. coli*: *Escherichia coli*; ICU: intensive care unit; UTI: urinary tract infection.

## Data Availability

Data is available upon reasonable request to the corresponding author.

## References

[1] Flores-Mireles A.L., Walker J.N., Caparon M., Hultgren S.J. (2015). Urinary tract infections: Epidemiology, mechanisms of infection and treatment options. Nat. Rev. Microbiol..

[2] Salam M., Al Anazi M., Al-Jeraisy M. (2015). Prevalence and predictors of antibiotic prescription errors in an emergency department, Central Saudi Arabia. Drug Healthc. Patient Saf..

[3] Alrashid S., Ashoor R., Alruhaimi S., Hamed A., Alzahrani S., Al Sayyari A. (2022). Urinary Tract Infection as the Diagnosis for Admission Through the Emergency Department: Its Prevalence, Seasonality, Diagnostic Methods, and Diagnostic Decisions. Cureus.

[4] E Silva A.C.S., Oliveira E.A. (2015). Update on the approach of urinary tract infection in childhood. J. Pediatr..

[5] Goossens H., Ferech M., Vander Stichele R., Elseviers M., ESAC Project Group (2005). Outpatient antibiotic use in Europe and association with resistance: A cross-national database study. Lancet.

[6] The World Health Report 2007: A Safer Future: Global Public Health Security in the 21st Century. https://apps.who.int/iris/handle/10665/43713?locale-attribute=ar&order=desc&scope=&sort_by=score&rpp=10&query=Theworldhealthreport2007:Asaferfuture:Globalpublichealthsecurityinthe21stcentury&search-result=true.

[7] Warren J.W., Abrutyn E., Hebel J.R., Johnson J.R., Schaeffer A.J., Stamm W.E. (1999). Guidelines for Antimicrobial Treatment of Uncomplicated Acute Bacterial Cystitis and Acute Pyelonephritis in Women. Clin. Infect. Dis..

[8] European Centre for Disease Prevention and Control, European Medicines Agency (2009). The Bacterial Challenge. Time to React: A Call to Narrow the Gap between Multidrug-RESISTANT bacteria in the EU and the Development of New Antibacterial Agents.

[9] Kumar V., George A., Viswanathakumar M. (2016). Study of clinical profile and risk factors associated with febrile urinary tract infection in preschool children. Int. J. Contemp. Pediatr..

[10] Lee S.J. (2015). Clinical Guideline for Childhood Urinary Tract Infection (Second Revision). Child. Kidney Dis..

[11] Antimicrobial Stewardship Subcommittee of the National Antimicrobial Resistance Committee and the General Administration of Pharmaceutical Care at Ministry of Health, Saudi Arabia (2018). National Antimicrobial Therapy Guidelines for Community and Hospital Acquired Infections in Adults. https://www.moh.gov.sa/en/CCC/healthp/regulations/Documents/National%20Antimicrobial%20%20Guidelines.pdf.

[12] Alamri A., Hassan B., Hamid M.E. (2021). Susceptibility of hospital-acquired uropathogens to first-line antimicrobial agents at a tertiary health-care hospital, Saudi Arabia. Urol. Ann..

[13] Balkhi B., Mansy W., Alghadeer S., Alnuaim A., AlShehri A., Somily A. (2018). Antimicrobial susceptibility of microorganisms causing Urinary Tract Infections in Saudi Arabia. J. Infect. Dev. Ctries..

[14] Almaiman L., Allemailem K.S., El-Kady A.M., Alrasheed M., Almatroudi A., Alekezem F.S., Elrasheedy A., Al-Megrin W.A., Alobaid H.M., Elshabrawy H.A. (2021). Prevalence and Significance of Pyuria in Chronic Kidney Disease Patients in Saudi Arabia. J. Pers. Med..

[15] Hameed T., Al Nafeesah A., Chishti S., Al Shaalan M., Al Fakeeh K. (2019). Community-acquired urinary tract infections in children: Resistance patterns of uropathogens in a tertiary care center in Saudi Arabia. Int. J. Pediatr. Adolesc. Med..

[16] Kader A.A., Angamuthu K. (2005). Extended-spectrum beta-lactamases in urinary isolates of *Escherichia coli, Klebsiella pneumoniae* and other gram-negative bacteria in a hospital in Eastern Province, Saudi Arabia. Saudi Med. J..

[17] Alzahrani M.A., Sadoma H.H.M., Mathew S., Alghamdi S., Malik J.A., Anwar S. (2021). Retrospective Analysis of Antimicrobial Susceptibility of Uropathogens Isolated from Pediatric Patients in Tertiary Hospital at Al-Baha Region, Saudi Arabia. Healthcare.

[18] Alasmary M.Y. (2021). Antimicrobial Resistance Patterns and ESBL of Uropathogens Isolated from Adult Females in Najran Region of Saudi Arabia. Clin. Pract..

[19] Bamshmous Y.S., Alwagdani S.M., Albarakati M.M., Alkouwait M.J., AlThwebi S.M. (2021). Infection with Gram-negative Bacteria among Children at a Tertiary Hospital in Jeddah, Saudi Arabia. Saudi J. Kidney Dis. Transplant..

[20] Garout W.A., Kurdi H.S., Shilli A.H., Kari J.A. (2015). Urinary tract infection in children younger than 5 years. Saudi Med. J..

[21] Alshamsan L., Al Harbi A., Fakeeh K., Al Banyan E. (2009). The value of renal ultrasound in children with a first episode of urinary tract infection. Ann. Saudi Med..

[22] Al-Ibrahim A.A., Girdharilal R.D., Akhter M., Jalal C., Alghamdy A.H., Ghazal Y.K. (2002). Urinary Tract Infection and Vesicouretral Reflux in Saudi Children. Saudi J. Kidney Dis. Transpl..

[23] Al-Harthi A.A., Al-Fifi S.H. (2008). Antibiotic resistance pattern and empirical therapy for urinary tract infections in children. Saudi Med. J..

[24] al Johani S.M., Akhter J., Balkhy H., El-Saed A., Younan M., Memish Z. (2010). Prevalence of antimicrobial resistance among gram-negative isolates in an adult intensive care unit at a tertiary care center in Saudi Arabia. Ann. Saudi Med..

[25] Akbar D.H. (2001). Urinary tract infection. Diabetics and non-diabetic patients—PubMed. Saudi Med. J..

[26] Kader A.A., Nasimuzzaman M. (2001). Antimicrobial Resistance Patterns of Gram-Negative Bacteria Isolated from Urine Cultures in Almana General Hospital. Ann. Saudi Med..

[27] Alamri A., Hamid E.M., Abid M., Alwahhabi A.M., Alqahtani K.M., Alqarni M.S., Abomughaid M. (2018). Trend analysis of bacterial uropathogens and their susceptibility pattern: A 4-year (2013–2016) study from Aseer region, Saudi Arabia. Urol. Ann..

[28] Alotaibi B.S., Tantry B.A., Farhana A., Alammar M.A., Shah N.N., Mohammed A.H., Wani F., Bandy A. (2023). Resistance Pattern in Mostly Gram-negative Bacteria Causing Urinary Tract Infections. Infect. Disord. Drug Targets.

[29] Alsohaim S.I.A., Bawadikji A.A., Elkalmi R., Mahmud M.I.A.D.M., Hassali M.A. (2019). Relationship Between Antimicrobial Prescribing and Antimicrobial Resistance Among UTI Patients at Buraidah Central Hospital, Saudi Arabia. J. Pharm. Bioallied Sci..

[30] Ahmed S.S., Shariq A., Alsalloom A.A., Babikir I.H., Alhomoud B.N. (2019). Uropathogens and their antimicrobial resistance patterns: Relationship with urinary tract infections. Int. J. Health Sci..

[31] Al-Helali N.S., Al-Asmary S.M., Abdel-Fattah M.M., Al-Jabban T.M., Al-Bamri A.-L.M. (2004). Epidemiologic study of nosocomial urinary tract infections in Saudi military hospitals. Infect. Control Hosp. Epidemiol..

[32] Alanazi M.Q. (2018). An evaluation of community-acquired urinary tract infection and appropriateness of treatment in an emergency department in Saudi Arabia. Ther. Clin. Risk Manag..

[33] Alanazi M.Q., Alqahtani F.Y., Aleanizy F.S. (2018). An evaluation of *E. coli* in urinary tract infection in emergency department at KAMC in Riyadh, Saudi Arabia: Retrospective study. Ann. Clin. Microbiol. Antimicrob..

[34] Sirkhazi M., Sarriff A., Aziz N.A., Almana F., Arafat O., Shorman M. (2014). Bacterial Spectrum, Isolation Sites and Susceptibility Patterns of Pathogens in Adult Febrile Neutropenic Cancer Patients at a Specialist Hospital in Saudi Arabia. World J. Oncol..

[35] Alghamdi S. (2021). Microbiological profile and antibiotic vulnerability of bacterial isolates from cancer patients. Cell. Mol. Biol..

[36] Bazaid A.S., Saeed A., Alrashidi A., Alrashidi A., Alshaghdali K., Hammam A.S., Alreshidi T., Alshammary M., Alarfaj A., Thallab R. (2021). Antimicrobial Surveillance for Bacterial Uropathogens in Ha’il, Saudi Arabia: A Five-Year Multicenter Retrospective Study. Infect. Drug Resist..

[37] Alqasim A., Jaffal A.A., Alyousef A.A. (2018). Prevalence of multidrug resistance and extended-spectrum β -Lactamase carriage of clinical uropathogenic *Escherichia coli* isolates in Riyadh, Saudi Arabia. Int. J. Microbiol..

[38] Al-Agamy M.H., Shibl A.M., Hafez M.M., Al-Ahdal M.N., Memish Z.A., Khubnani H. (2014). Molecular characteristics of extended-spectrum β-lactamase-producing *Escherichia coli* in Riyadh: Emergence of CTX-M-15-producing *E. coli* ST131. Ann. Clin. Microbiol. Antimicrob..

[39] Kader A.A., Kumar A., Dass S.M. (2004). Antimicrobial Resistance Patterns of Gram-Negative Bacteria Isolated from Urine Cultures at a General Hospital. Saudi J. Kidney Dis. Transplant..

[40] El-Bashier A.M. (1991). Bacteriuria, Incidence, Causative Microorganism, and Susceptibility Pattern at Qatif Central Hospital. Ann. Saudi Med..

[41] Kader A.A., Kumar A. (2005). Prevalence and antimicrobial susceptibility of extended-spectrum β-lactamase-producing Escherichia coli and Klebsiella pneumoniae in a general hospital. Ann. Saudi Med..

[42] Al-Asmary S.M., Al-Helali N.S., Abdel-Fattah M.M., Al-Jabban T.M., Al-Bamri A.-L.M. (2004). Infection Control Unit (Al-Bamri), Al-Hada Armed Forces Hospital, Taif, Kingdom of Saudi Arabia. www.smj.org.sa.

[43] Eltahawy A.T., Khalaf R.M.F. (1988). Urinary Tract Infection at a University Hospital in Saudi Arabia: Incidence, Microbiology, and Antimicrobial Susceptibility. Ann. Saudi Med..

[44] Abduljabbar H., Moumena R.A., Mosli H.A., Khan A.S., Warda A. (1991). Urinary Tract Infection in Pregnancy. Ann. Saudi Med..

[45] Balkhy H.H., Cunningham G., Chew F.K., Francis C., Al Nakhli D.J., Almuneef M.A., Memish Z.A. (2006). Hospital- and community-acquired infections: A point prevalence and risk factors survey in a tertiary care center in Saudi Arabia. Int. J. Infect. Dis..

[46] Al-Rubeaan K.A., Moharram O., Al-Naqeb D., Hassan A., Rafiullah M.R.M. (2012). Prevalence of urinary tract infection and risk factors among Saudi patients with diabetes. World J. Urol..

[47] Abdulmutalib D.A., Abato A.T., Mazi W., Senok A. (2013). P017: Reduction of catheter associated urinary tract infections following removal of unnecessary urinary catheters in a tertiary care hospital in Saudi Arabia. Antimicrob. Resist. Infect. Control.

[48] Al-Tawfiq J.A., Amalraj A., Memish Z.A. (2013). Reduction and surveillance of device-associated infections in adult intensive care units at a Saudi Arabian hospital, 2004–2011. Int. J. Infect. Dis..

[49] Hossain M.A., Mohal S., Islam M.S., Yusuf M.A. (2013). Prevalence of Ciprofloxacin Resistance among Gram-Negative Bacilli Isolated From Urinary Tract Infection Specimens at a Specialist Hospital in Riyadh, Saudi Arabia. J. Sci. Found..

[50] Alharthi A.A., Taha A.A., Edrees A.E., Elnawawy A.N., Abdelrahman A.H. (2014). Screening for urine abnormalities among preschool children in western Saudi Arabia. Saudi Med. J..

[51] El-Kersh T.A., Marie M.A., Al-Sheikh Y.A., Al-Kahtani S.A. (2015). Prevalence and risk factors of community-acquired urinary tract infections due to ESBL-producing Gram negative bacteria in an Armed Forces Hospital in Sothern Saudi Arabia. Glob. Adv. Res. J. Med. Med. Sci..

[52] Faidah H.S., Ashshi A.M., El-Ella G.A.A., Al-Ghamdi A.K., Mohamed A.M. (2015). Urinary Tract Infections among Pregnant Women in Makkah, Saudi Arabia. Biomed. Pharmacol. J..

[53] Al Yousef S.A., Younis S., Eman F., Moussa H.S., Bayoumi F.S., Ali A.M. (2016). Clinical and Laboratory Profile of Urinary Tract Infections Associated with Extended Spectrum β-Lactamase Producing *Escherichia coli* and *Klebsiella pneumoniae*. Ann. Clin. Lab. Sci..

[54] Kabbani M.S., Ismail S.R., Fatima A., Shafi R., Idris J.A., Mehmood A., Singh R.K., Elbarabry M., Hijazi O., Hussein M.A. (2016). Urinary tract infection in children after cardiac surgery: Incidence, causes, risk factors and outcomes in a single-center study. J. Infect. Public Health.

[55] Al-Mijalli S.H. (2017). Bacterial Uropathogens in Urinary Tract Infection and Antibiotic Susceptibility Pattern in Riyadh Hospital, Saudi Arabia. Cell. Mol. Med..

[56] Al-Hameed F.M., Ahmed G.R., Alsaedi A.A., Bhutta M.J., Al-Hameed F.F., Alshamrani M.M. (2018). Applying preventive measures leading to significant reduction of catheter-associated urinary tract infections in adult intensive care unit. Saudi Med. J..

[57] Gaid E., Assiri A., McNabb S., Banjar W. (2017). Device-associated nosocomial infection in general hospitals, Kingdom of Saudi Arabia, 2013–2016. J. Epidemiol. Glob. Health.

[58] al Wutayd O., al Nafeesah A., Adam I., Babikir I.H. (2018). The antibiotic susceptibility patterns of uropathogens isolated in Qassim, Saudi Arabia. J. Infect. Dev. Ctries..

[59] Albalawi S., Albalawi B., al Shwameen M.O., Alharbi M. (2018). Bacterial Susceptibility to Antibiotics in Urinary Tract Infections in Children, KSAFH, Saudi Arabia, Tabuk. Egypt. J. Hosp. Med..

[60] Taher I., Almaeen A., Aljourfi H., Bohassan E., Helmy A., Elmasry E., Saleh B., Aljaber N. (2019). Surveillance of antibiotic resistance among uropathogens in Aljouf region northern Saudi Arabia. Iran. J. Microbiol..

[61] Alshamrani M.M., El-Saed A., Alsaedi A., El Gammal A., Al Nasser W., Nazeer S., Balkhy H.H. (2019). Burden of healthcare-associated infections at six tertiary-care hospitals in Saudi Arabia: A point prevalence survey. Infect. Control. Hosp. Epidemiol..

[62] Ahmed N.J., Haseeb A., Elazab E.M., Kheir H.M., Hassali A.A., Khan A.H. (2021). Incidence of Healthcare-Associated Infections (HAIs) and the adherence to the HAIs’ prevention strategies in a military hospital in Alkharj. Saudi Pharm. J..

[63] Alrasheedy M., Abousada H.J., Abdulhaq M.M., Alsayed R.A., Alghamdi K.A., Alghamdi F.D., Al Muaibid A.F., Ajjaj R.G., Almohammadi S.S., Almohammadi S.S. (2021). Prevalence of urinary tract infection in children in the kingdom of Saudi Arabia. Arch. Ital. Urol. Androl..

[64] Aabed K., Moubayed N., Alzahrani S. (2021). Antimicrobial resistance patterns among different *Escherichia coli* isolates in the Kingdom of Saudi Arabia. Saudi J. Biol. Sci..

[65] Al-Mijalli S.H.S., Shami A.Y., Al-Salem R.A., Alnafisi N.M. (2022). Development of Diagnostic Capabilities for Complications of Bacterial Infection in Diabetic Patients. Rev. Diabet.Stud..

[66] Saleem M., Khaja A.S.S., Hossain A., Alenazi F., Said K.B., Moursi S.A., Almalaq H.A., Mohamed H., Rakha E., Mishra S.K. (2022). Catheter-Associated Urinary Tract Infection in Intensive Care Unit Patients at a Tertiary Care Hospital, Hail, Kingdom of Saudi Arabia. Diagnostics.

[67] Aldecoa Y.S., Alanazi A., Saleh G.B., Alshanbari N., Humayun T., Alsheddi F., El-Saed A., Alqahtani M., Alanazi K.H. (2022). Rates of urinary catheter-associated urinary tract infection in Saudi MOH hospitals: A 2-year multi-centre study. Int. J. Infect. Control..

[68] Abalkhail A., AlYami A.S., Alrashedi S.F., Almushayqih K.M., Alslamah T., Alsalamah Y.A., Elbehiry A. (2022). The Prevalence of Multidrug-Resistant *Escherichia coli* Producing ESBL among Male and Female Patients with Urinary Tract Infections in Riyadh Region, Saudi Arabia. Healthcare.

[69] Gupta K., Hooton T.M., Naber K.G., Wullt B., Colgan R., Miller L.G., Moran G.J., Nicolle L.E., Raz R., Schaeffer A.J. (2011). International Clinical Practice Guidelines for the Treatment of Acute Uncomplicated Cystitis and Pyelonephritis in Women: A 2010 Update by the Infectious Diseases Society of America and the European Society for Microbiology and Infectious Diseases. Clin. Infect. Dis..

[70] Rowe T.A., Juthani-Mehta M. (2013). Urinary tract infection in older adults. Aging Health.

[71] Shapiro D.J., Hicks L.A., Pavia A.T., Hersh A.L. (2014). Antibiotic prescribing for adults in ambulatory care in the USA, 2007–2009. J. Antimicrob. Chemother..

[72] Martínez M.A., Inglada L., Ochoa C., Villagrasa J.R., The Spanish Study Group on Antibiotic Treatments (2007). Assessment of antibiotic prescription in acute urinary tract infections in adults. J. Infect..

[73] Habak P.J., Griggs R.P. (2022). Urinary Tract Infection In Pregnancy.

[74] Al-Asmary S.M., Al-Helali N.S., Abdel-Fattah M.M. (2004). Nosocomial urinary tract infection Risk factors, rates and trends. Saudi Med. J..

[75] Flores-Mireles A., Hreha T.N., Hunstad D.A. (2019). Pathophysiology, Treatment, and Prevention of Catheter-Associated Urinary Tract Infection. Top. Spinal Cord Inj. Rehabil..

[76] Kandeel A. (2014). Prevalence and risk factors of extended-spectrum β-lactamases producing Enterobacteriaceae in a general hospital in Saudi Arabia. J. Microbiol. Infect. Dis..

[77] Obaid N.A. (2021). Preventive Measures and Management of Catheter-Associated Urinary Tract Infection in Adult Intensive Care Units in Saudi Arabia. J. Epidemiol. Glob. Health.

